# Detection and genetic characterization of Seoul Virus from commensal brown rats in France

**DOI:** 10.1186/1743-422X-11-32

**Published:** 2014-02-20

**Authors:** Tatiana Dupinay, Kieran C Pounder, Florence Ayral, Maria-Halima Laaberki, Denise A Marston, Sandra Lacôte, Catherine Rey, Fabienne Barbet, Katja Voller, Nicolas Nazaret, Marc Artois, Philippe Marianneau, Joel Lachuer, Anthony R Fooks, Michel Pépin, Catherine Legras-Lachuer, Lorraine M McElhinney

**Affiliations:** 1 Université de Lyon, VetAgro Sup, USC 1233/Equipe « Pathogènes émergents et rongeurs sauvages (PERS), F-69280 Marcy-L’Etoile, France; 2 Institute of Integrative Biology, University of Liverpool, Crown Street, Liverpool L69 7ZB, UK; 3 Wildlife Zoonoses and Vector-borne Diseases Research Group, Animal Health and Veterinary Laboratories Agency (AHVLA), New Haw, Addlestone, Surrey KT15 3NB, UK; 4 Anses-Laboratoire de Lyon, Unité Virologie, F-69347 Lyon, France; 5 Viroscan 3D / Profilexpert, Faculté de Médecine et de Pharmacie, 3453 CNRS-US7 Inserm, 8 avenue Rockefeller, aile C2, 69373, Lyon Cedex 08, France; 6 National Consortium for Zoonosis Research, University of Liverpool, Leahurst, Neston, South Wirral CH64 7TE, UK

**Keywords:** Hantavirus, SEOV, France, Brown rat, *Rattus norvegicus*, Next generation sequencing, Viral enrichment

## Abstract

**Background:**

Hantaviruses are single-stranded RNA viruses, which are transmitted to humans primarily via inhalation of aerosolised virus in contaminated rodent urine and faeces. Whilst infected reservoir hosts are asymptomatic, human infections can lead to two clinical manifestations, haemorrhagic fever with renal syndrome (HFRS) and hantavirus cardiopulmonary syndrome (HCPS), with varying degrees of clinical severity. The incidence of rodent and human cases of Seoul virus (SEOV) in Europe has been considered to be low, and speculated to be driven by the sporadic introduction of infected brown rats (*Rattus norvegicus*) via ports.

**Methods:**

Between October 2010 and March 2012, 128 brown rats were caught at sites across the Lyon region in France.

**Results:**

SEOV RNA was detected in the lungs of 14% (95% CI 8.01 – 20.11) of brown rats tested using a nested pan-hantavirus RT-PCR (polymerase gene). Phylogenetic analysis supports the inclusion of the Lyon SEOV within Lineage 7 with SEOV strains originating from SE Asia and the previously reported French & Belgian SEOV strains. Sequence data obtained from the recent human SEOV case (Replonges) was most similar to that obtained from one brown rat trapped in a public park in Lyon city centre. We obtained significantly improved recovery of virus genome sequence directly from SEOV infected lung material using a simple viral enrichment approach and NGS technology.

**Conclusions:**

The detection of SEOV in two wild caught brown rats in the UK and the multiple detection of SEOV infected brown rats in the Lyon region of France, suggests that SEOV is circulating in European brown rats. Under-reporting and difficulties in identifying the hantaviruses associated with HFRS may mask the public health impact of SEOV in Europe.

## Introduction

Hantaviruses (family *Bunyaviridae*, genus Hantavirus) are single-stranded RNA viruses. Unlike other members of the *Bunyaviridae*, hantaviruses are not transmitted by arthropods but primarily by rodents of the families Cricetidae and Muridae, although insectivore and bat hosts have also been reported [1,2]. Each hantavirus appears to be adapted and largely restricted to an individual reservoir host species, implying that they have co-evolved, although phylogenetic analyses suggests that this apparent co-evolution may be more attributed to recent preferential host switching and local adaptation [3].

Transmission to humans is primarily via inhalation of aerosolised virus in contaminated rodent urine and faeces. Whilst infected reservoir hosts are asymptomatic, human infections can lead to two clinical manifestations, haemorrhagic fever with renal syndrome (HFRS) and hantavirus cardiopulmonary syndrome (HCPS), with varying degrees of morbidity and mortality [4]. Surveillance in Europe has detected six rodent-borne hantaviruses; Dobrava-Belgrade virus (DOBV), Saaremaa virus (SAAV), Seoul virus (SEOV), Puumala virus (PUUV), Tatenale virus (TATV) and Tula virus (TULV) plus two insectivore-borne hantaviruses; Seewis virus (SWSV) and Nova virus (NVAV) [4-7]. The relative geographic distribution of each hantavirus is defined by their reservoir host [7]. The most common and widespread hantavirus across northern, central and eastern Europe is PUUV, which is associated with the mildest form of HFRS [4].

Unlike other hantaviruses, SEOV has a global distribution due to the worldwide dispersal of its carrier host (*Rattus sp*). Confirmed human SEOV infections have been reported in Asia (Japan [8], South Korea [9], China [10,11]) and the Americas (USA [12], Brazil [13]). Norwegian/brown rats (*Rattus norvegicus*) are a cosmopolitan species and represent an emerging and widely distributed host of hantavirus in China, where, a total of 1,557,622 cases of HFRS were reported in humans between 1950–2007 with 46,427 deaths (3%) [11,14]. Historically, the presence of Seoul virus in Europe was considered anecdotal and speculated to be driven by the sporadic introduction of infected brown rats via ports [4]. Previously, a single HFRS case near the port city of Lyon, France, had only been confirmed serologically by SEOV FRNT [15] and SEOV antibodies had been reported in brown rats in France (10-78.9%) and Belgium (27.1%) [15,16]. However, more recently the virus has been isolated from wild brown rats in the UK [17] and pet rats in the UK and Sweden [18-20]. In addition, SEOV associated HFRS has been reported in four cases in the UK and France, all of which were clinically severe and involved renal impairment [17,21,22].

This study aimed to determine the presence of SEOV in wild rats (*R. norvegicus*) trapped in and around Lyon, France and analyse any resulting molecular epidemiological data. The study also determined the optimal approach to obtaining SEOV genomic sequence data directly from infected lung tissue by comparing different sample preparation techniques and next generation sequencing (NGS) platforms.

## Results and discussion

### Detection of SEOV virus

A total of 128 brown rats were caught from 23 sites in and around Lyon. Seoul hantavirus RNA was detected in 14% (18/128) of the rat lung samples tested in triplicate (95% CI 8.01 – 20.11). Positive rats were detected in 6 out of the 23 sites of capture (see Additional file 1).

There was a male bias of 2:1 in the infected individuals: 11 adult males, one juvenile male, one pregnant female and five adult females. The proportion of all males infected was larger than females, 16.4% and 11.3% respectively, but this was not significant (Pearson’s Chi-squared test, χ2 = 0.6568, df = 1, P = 0.4177).

A male biased ratio amongst SEOV infected rats is not uncommon, and has been reported on several occasions [23-25]. Whilst neither male nor female rats are believed to be more susceptible to Seoul virus infection, males do shed the virus for a longer duration in their urine, faeces and saliva [25] and so the viral RNA may be detectable for longer in the host tissues. In addition, the primary route of transmission between adult males is thought to be through wounds [26], so it has been suggested that the likelihood of males acquiring the Seoul virus is greater due to them having more aggressive encounters [25].

**Figure 1:**
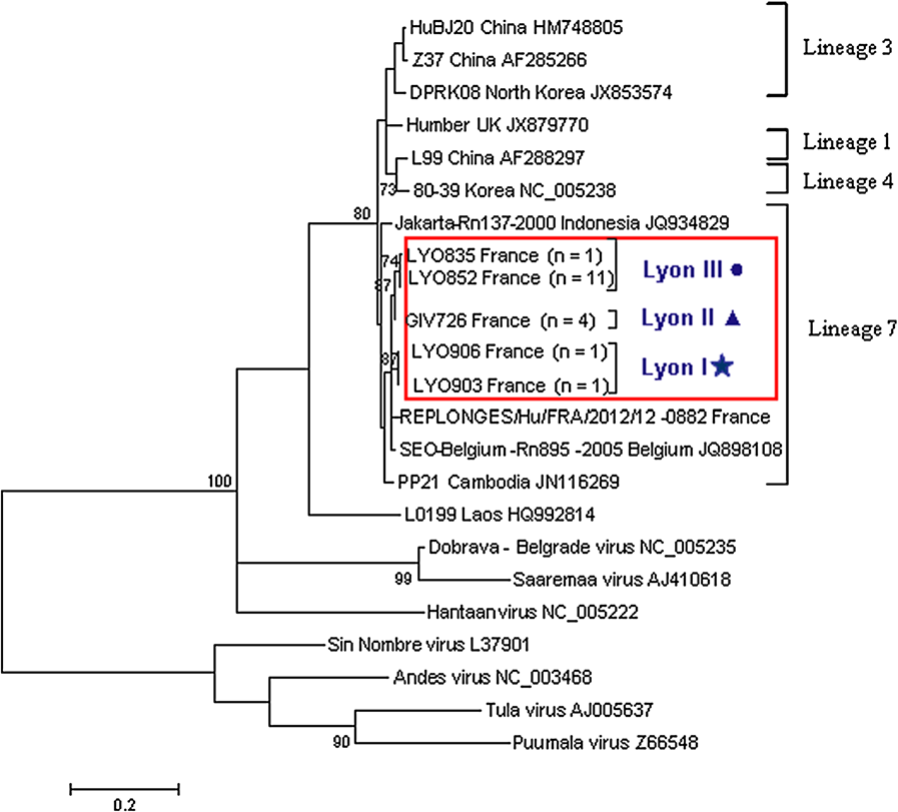
**Maximum likelihood tree using the model T92 + Gamma **[28]**for SEOV partial L segment sequences n = 23 in MEGA5 **[35]**.** The trees are drawn to scale, with branch lengths measured in the number of substitutions per site. The scale bar indicates nucleotide substitutions per site. Only bootstrap support of >70% are shown. Positions with less than 95% site coverage were eliminated. There were a total of 317 positions in the final dataset. The phylogenetic positions of groups Lyon I, II and III are shown in relation to representative Seoul strains (identical sequences removed for clarity). GIV726 partial L sequence was identical to GIV733, GIV737 and GIV757 (Lyon II). LYO852 partial L sequence was identical to LYO799, 837, 838, 839, 843, 845, 848, 853, 884 and 871 (Lyon III). Genbank accession numbers are shown next to taxa names.

All 18 RT-PCR positive rats were selected for genetic analysis and partial sequences of the L segment (317 bp) were recovered. Eight variable sites were located within this partial sequence. Phylogenetic analysis resolved the Lyon SEOV into three clusters (Lyon I, II and III; Figure 1, Additional file 1) reflecting their disparate trapping locations. The Lyon I, II and III variants were detected at 1 (n = 2 rats), 2 (n = 4 rats) and 3 (n = 12) locations respectively (See Additional file 2A). No co-circulation of variants was observed. Lyon I and II were geographically restricted, whereas Lyon III was the most frequently detected and widespread of the variants (Additional file 1). All Lyon SEOV partial L sequences, including that derived from the recent human case (Replonges) showed highest identity to the Belgium SEO/Belgium/Rn895/2005 strain (JQ898108). Lyon I partial L sequences (LYO903 and LYO906) were more divergent from the other Lyon sequences (0.5-1.4%), but they were the closest Lyon SEOV to the nearby severe HFRS case in Replonges (~97.8%) [21]. All 18 RT-PCR L sequences clustered with previously described Lineage 7 sequences within Phylogroup A [14,27], with moderate bootstrap support. Despite their disparate isolation, most SEOV variants published to date are genetically homogenous [11,14] making it difficult to determine the source of introduction. However, at a local level the higher degree of sequence homology can result in geographical clustering as observed in China [14], the UK [18] and for the Lyon SEOV in this study. To further study the molecular epidemiology of a Lyon SEOV strain in the context of global SEOV, we obtained full genome sequence of a representative sample. The strong and non-degraded SEOV positive lung tissue sample LYO852 was chosen as it represented the most frequent and widespread variant detected (Lyon III).

Due to the low abundance of viral sequences relative to total host nucleic acids, we optimised the procedure to obtain complete SEOV genome using two next generation sequencing platforms and differing sample preparation approaches.

### Roche 454 output and assembly statistics

Viral specific reads from LYO852 were obtained directly from lung tissue on the Roche 454 NGS platform without the use of viral enrichment or ultra centrifugation. *De Novo* assembly of the 454 reads yielded 59 contigs (consisting of 73,105 reads, totalling 24,730,464 bp) representing 82% total reads, with a mean length of 702 bp (ranging between 105-2920 bp). There were 15 contigs ≥500 bp. Based on BLAST identity searches, all contigs were host or mycoplasma sequences. Mapping of the reads using GS Reference Mapper (Roche) with published SEOV genome sequences identified 44 (0.03%) SEOV specific reads yielding 9 contigs in total for LYO852. Two partial nucleocapsid (S) gene contigs were retrieved of 715 and 786 bp. Three partial glycoprotein (M) gene contigs were retrieved of 612, 987 and 1,735 bp. Four partial polymerase (L) gene contigs were retrieved, of 459, 603, 740 and 1,564 bp. Following alignment, the total 454 coverage for each of the three segments of LYO852 was 84.8% (S), 91.3% (M) and 51.5% (L).

### Viral enrichment methodology

**Figure 2:**
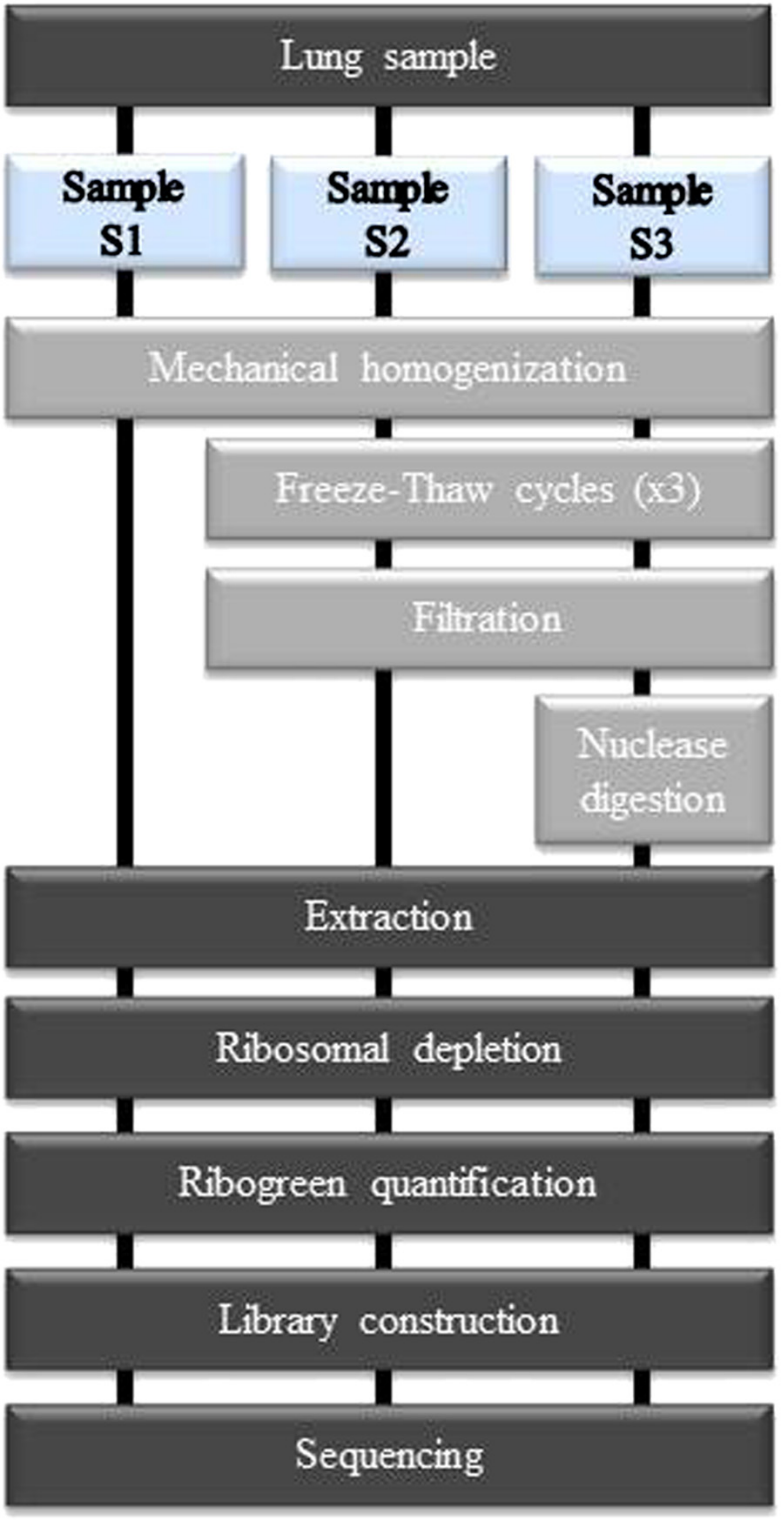
**Workflow for the preparation of lungs tissue samples for next generation sequencing.** All samples were extracted using RNeasy mini kit (Qiagen) and treated by ScriptSeq complete golg kit (Epicentre) and submitted to Illumina sequencing. Comparison of viral enrichment methods: no enrichment procedure was performed for the S1 condition, a filtration step was included for S2 condition and a filtration step with nuclease digestion were combined for the condition S3.

To improve upon the genome coverage obtained using the Roche-454 platform, we compared several purification procedures and employed the Illumina NGS platform (Figure 2). We combined the homogenization step with or without freeze-thaw cycles, with or without sample filtration (to remove cells and mitochondria), and with or without either of 2 nuclease digestion protocols (to degrade DNA and RNA host contaminants) (data not shown). We observed that the nuclease digestion for 90 min was not sufficient to remove all rRNA and therefore we performed a ribosomal depletion. We compared the various approaches using qPCR assays for GAPDH, β-actin cDNA and viral RNA (data not shown) in comparison to the non-enriched sample (S1). The two optimal enrichment protocols involved homogenisation of the tissue with a micropestle in cold HBSS followed by dry ice freeze thaw cycles and a centrifugation/filtration step, without (S2) or with a subsequent 2 step-digestion (S3) (Figure 2). The 3 resultant samples (S1, S2 and S3) were then used to carry out next-generation sequencing using the Illumina platform.

### Illumina outputs and assembly statistics

Without viral enrichment (S1), 513 Illumina contigs were generated for a total length of 189,884 bp. There were 31 contigs ≥1000 bp. Of all the contigs, 13 corresponded to SEOV genome (0.34%).

Illumina sequencing for each of the three samples generated between 62 and 91 million sequence reads but 17% to 29% of the reads were discarded after quality filtering (Table 1). As shown in Table 1, the sample S1 presented a larger number of reads that aligned with the reference rat genome sequence (88%) than for the virus enriched samples S2 and S3 (approximately 71 and 47% respectively). Notably, the viral reads were 6 times more abundant in the S3 sample (2.20%) in comparison to the S1 sample (0.34%). Furthermore, the S3 sample appeared more enriched in SEOV reads than the two other samples (Table 1) and that obtained using the Roche 454 platform. Mapping sequence reads revealed complete or near complete (>99%) coverage of the SEOV reference genomes for the virally enriched samples (S3). Complete SEOV genome sequences were recovered from the LYO852 sample and the SEOV consensus sequences of the three samples were identical. The SEOV sequences have been deposited in GenBank under accession numbers KF387723 to KF387725. Hence, we report the significantly improved chances of successfully obtaining complete viral genome sequences by NGS following simple viral enrichment steps. The S3 enrichment approach will be assessed for future NGS analysis on the Roche-454 platform.

**Table 1:** Overview of the sequence reads and mapped SEOV sequences obtained using the Illumina NGS platform for sample preparations S1-S3

**Illumina Sample Prep**	**Read data**							**Reads from SEOV Segments**			**Reads from SEOV**	**% reads from SEOV**
	**Total reads**	**PF**	**PF aligned**	**% PF aligned**	**% Ribosomal**	**Viral reads**	**% viral reads**	**Segment S**	**Segment M**	**Segment L**		
**S1**	91,661,298	75,522,658	66,580,460	88.16%	0.99%	255,911	0.34%	12,675	30,211	13,617	56,503	22%
**S2**	83,920,361	59,434,134	42,659,876	71.78%	30.95%	434,756	0.73%	10,610	37,111	43,345	91,066	20.90%
**S3**	62,479,875	46,105,731	21,974,935	47.66%	21.55%	1,016,600	2.20%	112,152	363,008	381,521	856,681	84.29%

PF: Pass Filter (Quality Check). PF Aligned – sequences aligned with rat genome (see methods).

### Genetic and phylogenetic analysis

We report the complete genomic sequence of a SEOV strain isolated from *R. norvegicus* in France.

The S-segment has a total of 1755nt with a deduced coding sequence of 1290nt. The putative encoded nucleoprotein (N) (AGZ59811) is 429 amino acids for a predicted 48KDa protein. The S-segment complete coding sequence shared the highest identity (98%) with the complete coding sequences of Vietnamese strains [29,30], the two Singaporean strains Rn41 and Rn46 [31], the SEOV Belgian Rn895 strain and the French Replonges strain [21] (respective GenBank accession numbers: AB618112 to AB618126, GQ274944 and GQ274945, JQ898106 and KC902522). All these strains originated from wild *R. norvegicus* but the Replonges strain was obtained from a patient. The putative N protein was identical to the deduced N protein of strains that originated from South Korea, China, Vietnam, the United Kingdom and the French Replonges strain (NP_942556, ADE34611, BAL46798, AGB05597 and AGL45258 respectively). It is identical to the 91 amino acid long partial sequence of the France 90 strain (CAI47594), implying that the 2 substitutions at the nucleotide level were silent. It also presents 99.76% nucleotide identity and 100% amino acid similarity with the complete coding region of Belgium/Rn895 strain (AFN11574).

The M-segment sequence (3638nt long) has a deduced coding sequence of 3402nt encoding for a putative polyprotein of 125KDa (AGZ59810). This putative protein precursor presented the conserved cleavage site (WAASA) that is required to give rise to the Gn and Gc membrane glycoproteins [32]. The full M-segment sequence shared, at the nucleotide and protein levels, the highest identity with the strains originating from Vietnam and Singapore. The putative polyprotein is identical to the predicted partial protein sequence of the France90 strain (CAI47595) confirming that the 3 substitutions are silent. It also presents 6 substitutions (5 of which are synonymous substitutions) when compared with the sequence of the Belgium/Rn895 strain (JQ898107, AFN11575).

The L-segment sequence obtained is 6511nt long. The deduced coding sequence (6456nt) encodes the putative RNA-dependent RNA polymerase (AGZ59809) whose size is predicted at 246KDa. When compared exclusively to full length coding sequences, the LYO852 strain shared the highest identity with the L-segment of the China Z37 strain (96%). However, when considering partial sequences, it presented the highest identity (99%) with the Belgium/Rn895 strain partial L-segment sequence (JQ898108).

**Figure 3:**
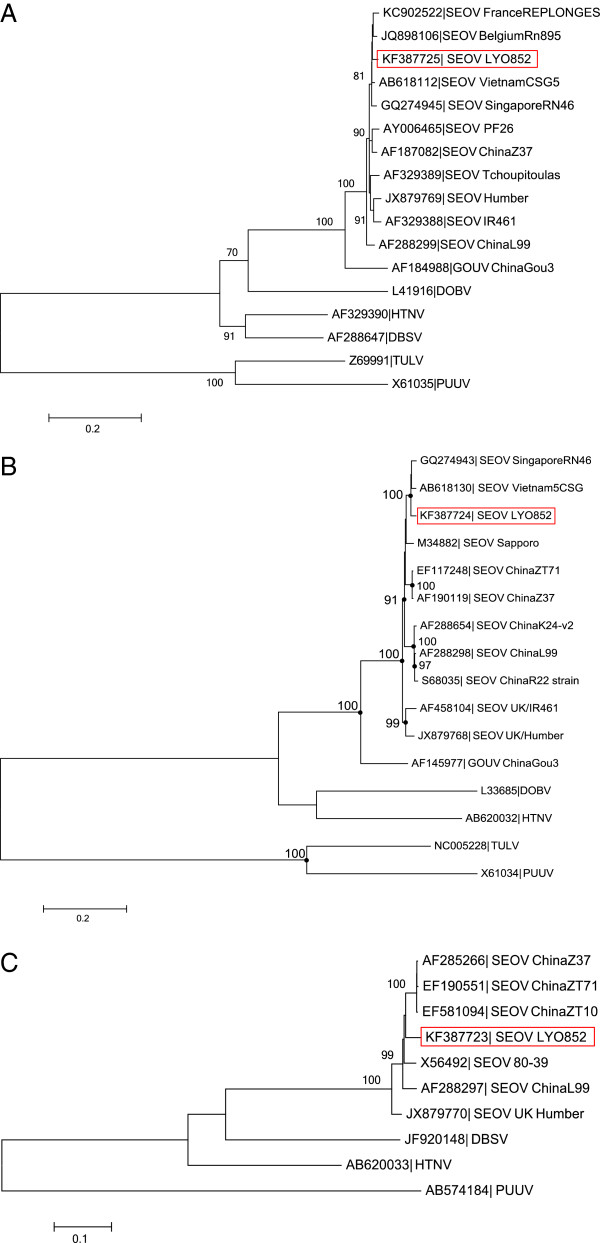
**Phylogenetic tree of hantaviruses based on the S, M and L segment sequences (complete coding region).** All analyses were performed using the MEGA software [35]. **A**. S segment: analysis was performed applying the generalized time reversible model (GTR) using a Gamma distribution with five rate categories and invariant site (+G + I) Only bootstrap percentages ≥70% (from 1000 resamplings) are indicated. **B**. M segment: analysis was performed applying the generalized time reversible model (GTR) using a Gamma distribution with five rate categories (+G). For clarity purpose, the nodes corresponding to bootstrap percentages ≥70% (from 1000 resamplings) are indicated by dots. **C**. L segment: analysis was performed applying the generalized time reversible model (GTR) using a Gamma distribution with five rate categories (+G). Only bootstrap percentages ≥70% (from 500 resamplings) are indicated. The scale bars indicate nucleotide substitution per site. The red boxes highlight the LYO852 strain described in the present study. Accession numbers are indicated for each strain in the corresponding taxa name.

From the 3 reconstructed phylogenetic trees (Figure 3), the LYO852 strain is resolved in the SEOV clade within the South East Asian virus lineage, also referred to as lineage 7 [27,33]. According to the nucleotide and protein analysis and the phylogenetic reconstruction of the full length S-segment, the LYO852 strain shared the most evolutionary relatedness with the strains previously detected either in Belgium (Belgium/Rn895) or in France (Replonges strain). Altogether, these findings are consistent with the earlier genetic description of the Belgium/Rn895 strain [33]. It supports further the hypothesis of a SEOV introduction in Europe due to the migration of its carrier, the brown rat, during trade between China and Europe [14]. However, the Lyon SEOV (LYO852) strain is clearly distinct from the strains isolated in the United Kingdom (IR461, Humber and Banbury strains) which appear to represent a distinct lineage. As the Replonges strain had been detected in a patient, our results raise concerns regarding the circulation of the SEOV virus in the Lyon area. Unfortunately, the wider distribution of SEOV in France is currently unknown. In particular, SEOV prevalence should be investigated in other large cities such as Marseille or Paris where commensal rodent populations are significant.

We report the presence of multiple foci of SEOV infected wild brown rats trapped in disparate locations in and around the large French city of Lyon. The recent detection of SEOV in wild brown rats in the UK [17] and pet rats in the UK and Sweden [18-20] may suggest SEOV emergence. However, there has been limited surveillance for hantaviral RNA in brown rats in Europe. It is likely that future surveillance will identify similar foci of infection in *R.Norvegicus* in other European countries. The contribution of SEOV to European HFRS cases should be further investigated to estimate the public health impact posed by commensal brown rats.

## Materials and methods

### Sample collection

Between October 2010 and March 2012, 128 brown rats *(Rattus norvegicus*) were trapped in and around the city of Lyon, France. Rats were trapped using small (28 cm × 9 cm × 9 cm) or large (50 cm × 15 cm × 15 cm) single catch rat traps. Captured rats were transported to the laboratory where live rats were immediately anaesthetised using Isofluorane and sacrificed by cervical dislocation. Each rat was aseptically dissected. Lung tissues were collected from different lobes.

Rats provided for this study were trapped for the purpose of pest control (agreement no. 69-1810). They were euthanized and used (agreement no. 69-020931) according to ethical rules supervised by the ethical committee of VetAgroSup and European regulation (Directive EU 86/609).

### Screening for Hantavirus RNA

Immediately after collection, lung tissue was stored at -80°C pending further analysis. Approximately 50 to 100 mg of lung tissue was homogenised in 1 ml TRIzol® Reagent (Invitrogen, Life Technologies, Paisley, UK) with QIAGEN Stainless steel beads (5 mm) using a QIAGEN TissueLyser (Qiagen, UK) for 2 mins at 30 Hz. RNA was extracted from the homogenate according to the manufacturer’s instructions (Invitrogen, Life Technologies, Paisley, UK). The RNA samples were reverse transcribed using random hexamers and screened for hantavirus as previously described [5] employing a pan-hantavirus nested RT-PCR directed against partial polymerase (L) gene sequences [34].

### Phylogenetic analysis of partial L gene sequences

Multiple nucleotide sequence alignments of the 18 partial polymerase gene sequences and available published SEOV sequences were generated in MEGA5 [35]. Sequence identities were compared using Geneious 5.6.5. Optimum substitution models were estimated and maximum likelihood phylogenetic trees generated in MEGA5 [35] with bootstrap replications of 10,000 [36].

### Roche-454 and Illumina platform sequencing

Initially, the Roche-454 was employed to obtain genome sequence for a representative Lyon SEOV following previous optimisation and success in obtaining complete genome coverage for lyssaviruses using this platform [37]. Viral specific reads from LYO852 were obtained directly from lung tissue on the Roche 454 platform without the use of viral enrichment or ultra centrifugation. Briefly, for performing 454 Roche sequencing, the TRIzol® extracted viral RNA obtained during hantavirus screening was depleted of host genomic DNA using RNase-free DNAse (Qiagen, UK) and host ribosomal RNA was depleted using Terminator™ 5’-Phosphate-Dependent Exonuclease (Epicentre Biotechnologies) as described previously [37]. The RNA was fragmented, a random-primed cDNA library was made and run using the Roche 454 GS FLX System. The sequencing data were initially assembled in the GS *de novo* assembly software (Roche). Subsequently, previously published SEOV sequences were used to map specific reads from the original raw data using GS Reference Mapper (Roche).

Subsequently Illumina sequencing was assessed. Total RNA extractions were performed using RNeasy mini kit (Qiagen) according to the manufacturer’s instructions. The remaining rRNA was depleted using ScriptSeq complete gold kit Human/Mouse/Rat (Epicentre) following manufacturer instructions and controlled with the 2100 Bioanalyzer using “Eukaryote total RNA Pico Assay” (Agilent Technologies). 3.5-5 ng of depleted RNA was prepared for NGS using the Illumina protocol where 10 cycles of PCR were performed and standard TruSeq adapters and TruSeq barcoded primers were used. A final size selection was performed by native agarose gel electrophoresis to yield a library of inserts 250-350 bases in length. The library was extracted from the agarose gel using purification columns. The prepared library was then loaded onto an Illumina HiSeq 2500 v3 single read flow cell, standard cluster generation was performed on a Cbot and sequenced for 50 bases.

### Sequence reads

Reads were processed using CASAVA 1.8.2 and demultiplexed based on index sequences. The FastQC was used for Quality Check. Sequences were aligned, first, using TopHat 2.0.6 to the Rat genome and the unaligned reads were aligned using Bowtie 2.0.2 software against known viruses.

### Viral enrichment and Illumina Hiseq 2500 v3 sequencing

The different approaches for virus enrichment were also evaluated in this study for the infected lung tissue sample (LYO852) (Figure 2). Briefly, a piece of lung tissue was immersed in 1 ml of HBSS 1X and homogenized with micropestle and then placed on dry ice for approximately two minutes until frozen, and thawed quickly before returning to ice. Homogenization followed by freezing and thawing was repeated a further two times to disrupt the cells. Samples were then spun at 1500×g for 5 minutes at 4°C to pellet the nuclei and large cellular aggregates. The resulting supernatant was transferred to a new tube and 2 different treatments were applied: for the condition S2 a step-wise filtration process involving 0.45 μm polyethersulfone membrane filters (diameter 13 mm) (Millipore) was performed before RNA extraction. Condition S3 combined a step-wise filtration and a 2-step digestion with 25U of RNase I at 37°C for 90 min in 1× RNase I buffer and DNA is removed on-column (Qiagen). Following treatment, we extracted viral encapsidated RNA and residual host nucleic acids using the RNeasy mini extraction kit (Qiagen). Viral RNA was eluted to a final volume of 30 μl. Total RNA concentration was quantified with Quant-iT ribogreen RNA kit (Invitrogen).

The remaining rRNA was depleted using ScriptSeq complete gold kit Human/Mouse/Rat (Epicentre) following the manufacturer’s instructions and quantified using the 2100 Bioanalyzer using “Eukaryote total RNA Pico Assay” (Agilent Technologies).

### Reverse transcription and qPCR quantification

cDNA was generated with random hexamers using the iScript™cDNA Synthesis kit (Bio-Rad). RNA (30 ng) from each sample was incubated in the presence of 5× iScript reaction mix (containing iScript Reverse transcriptase) and nuclease-free water added to bring the final reaction volume to 20 μl. This volume was incubated at 25°C for 5 min, at 42°C for 30 min, at 85°C for 5 min. To quantify the enrichment of viral RNA, we performed various real-time PCRs targeting cDNA of Seoul Hantavirus, GAPDH and β-actin (see Additional file 2 for primer details). We calculated the fold enrichment in viral RNAs by comparing the proportion of encapsidated viral RNA CT (threshold cycle) values between the control and each treatment.

### Genetic and phylogenetic analysis of genomic segments

The deduced amino acid sequences of the 3 genomic segments of the LYO852 strain were obtained using the Serial Cloner 2.6.1 software. The complete coding sequence of the S, M and L segments and the predicted protein sequences were compared to the NCBI database using the BLAST program (http://blast.ncbi.nlm.nih.gov/).

Multiple sequence alignments of coding sequences were carried out using ClustalW algorithm in the MEGA 5.2.2 software (default parameters) [35]. Phylogenetic reconstructions were performed using the Maximum Likelihood statistical method. Bootstrapping (1000 or 500 resamplings) was applied according to the best-fit substitution model recommended.

## Competing interests

All of the authors declare that they have no competing interests with respect to the publication of this manuscript.

## Authors’ contributions

TD, KCP, DAM and KV were involved in the PCR screening and NGS work. SL, CR, FB and NN were involved in viral enrichment and Illumina sequencing. MHL was involved in phylogenetic analysis. PM, MA, MP, ARF, JL, CLL and LMM were involved in project conception, data analysis and logistical support. FA coordinated the trapping and post-mortems of the rodents as part of the WildTech consortium (http://www.wildtechproject.com/wildtech/). All authors contributed to the writing of this manuscript. All authors read and approved the final manuscript.

## Supplementary Material

Additional file 1 **The locations of the trapping sites (circles) within a) France and b) Rhône-Alps department.** SEOV positive variants ‘Lyon I, II and III’ are represented by a star, triangle and blocked out circles, respectively. Click here for file

Additional file 2 **A-Distribution of the SEOV variants detected in 6 of the 23 sites sampled. B**-Oligonucleotides used in this study. vRNA: viral RNA. Click here for file
