# Supplementary figures and images for: Detection and genetic characterization of Seoul Virus from commensal brown rats in France
Source: Virol J. 2014 Feb 20;11:32. doi: 10.1186/1743-422X-11-32 (PMC3944734; doi:10.1186/1743-422X-11-32)

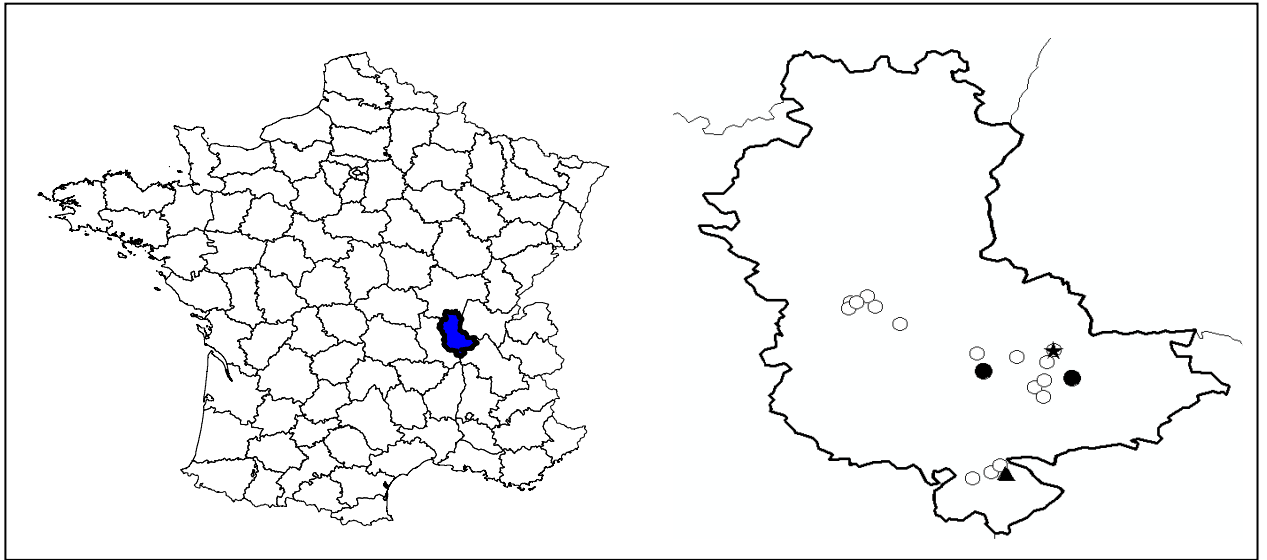

**Additional file 1**

Supplement: Additional file 1 — The locations of the trapping sites (circles) within a) France and b) Rhône-Alps department. SEOV positive variants ‘Lyon I, II and III’ are represented by a star, triangle and blocked out circles, respectively. [file 1743-422X-11-32-S1.pdf]
